# Impact of COVID-19 on the Destiny of Bariatric Patients

**DOI:** 10.3390/nu15010163

**Published:** 2022-12-29

**Authors:** Paola Gualtieri, Marco Marchetti, Laura Di Renzo, Gemma Lou De Santis, Roselisa Palma, Carmela Colica, Giulia Frank, Antonino De Lorenzo, Nicola Di Lorenzo

**Affiliations:** 1 Section of Clinical Nutrition and Nutrigenomics, Department of Biomedicine and Prevention, University of Tor Vergata, Via Montpellier 1, 00133 Rome, Italy; 2 School of Specialization in Food Science, University of Rome Tor Vergata, 00133 Rome, Italy; 3 PhD School of Applied Medical-Surgical Sciences, University of Rome Tor Vergata, Via Montpellier 1, 00133 Rome, Italy; 4 CNR, IBFM UOS, Università Magna Graecia, Viale Europa, 88100 Germaneto, Italy; 5 Department of Surgical Sciences, Università di Roma Tor Vergata, Via Montpellier 1, 00133 Rome, Italy

**Keywords:** bariatric surgery, metabolic surgery, obesity, COVID-19, SARS-CoV-2

## Abstract

Obese patients reported worse outcomes of COVID-19 related to prothrombotic and low-grade inflammation status. During the SARS-CoV-2 outbreak, all non-elective surgeries were postponed, including bariatric surgery (BS). This umbrella review wants to underline obesity as a condition provoking low-grade chronic inflammation, and increasing severe COVID-19 risk; to relaunch the prioritization of BS. The literature search was conducted in March 2022 via Pubmed (MEDLINE) and focused on reviews, systematic reviews, and meta-analyses published in peer-reviewed journals. Terms “bariatric surgery” OR “obesity surgery” OR “metabolic surgery” were analyzed with “COVID-19” OR “SARS-CoV-2” using the AND modifier. Only 13 studies of the 406 screened met the objective. The procrastination of BS over the past two years determined a delay in obesity treatment and severe consequences. The COVID-19 pandemic has had a huge impact on economic costs. Although BS has high costs, a lifetime cost advantage over conventional weight loss methods was demonstrated. As the pandemic continues, health policies must recognize obesity as a disease-predisposing factor for SARS-CoV-2 infection, considering COVID-19 as a new comorbidity mitigable by BS. Care pathways for obese patients in COVID/post-COVID era should be revitalized and the concept of elective surgery attributed to BS should be reformulated.

## 1. Introduction

Since December 2019, SARS-CoV-2 has been spreading worldwide until it became a pandemic [1].

In Italy, the first reported case was in February 2020, and in May, the amount was 211,938, with 29,079 deaths and probably about 5,000,000 infected people [2]. Obese patients reported worse outcomes, complications, and intensive care therapies due to the typical illness related to the prothrombotic status and low-grade inflammation of obesity, namely: insulin resistance, dyslipidemia, hypertension, atherosclerosis, and type 2 diabetes mellitus (T2DM) [3].

In 2016, according to the body mass index (BMI), 45% of adults worldwide were overweight or obese. The lockdown measures taken to limit the spreading of the virus have indeed increased this number because of the changes in eating habits and decreased physical activity [4]. The time spent at home is associated with an unhealthy diet and a sedentary lifestyle.

It is essential to describe the true meaning of obesity. Obesity is a very complex disease due to individual, environmental and genetic factors, which lead to an excessive accumulation of fat mass and is related to systemic inflammation [5]. Even though BMI is the most common parameter used to define obesity, as it is useful to describe a population, it lacks significance for a single patient because it does not consider body composition. It is known that the percentage of fat mass behind BMI is the real trigger of metabolic syndrome, insulin resistance, dyslipidemia, hypertension, and several other illnesses and chronic diseases [6]. Furthermore, a crucial role is played by the distribution of fat mass. Particularly, visceral adipose tissue (VAT) is associated with cardiovascular and metabolic risks [3], and it is clear that the positive relationship between bariatric surgery and weight loss improves cardiometabolic risks [7]. Moreover, the endocrine mechanisms of the chronic inflammatory response in adipose tissue favor infections. In obese patients, there is an increased synthesis of pro-inflammatory cytokines, hence the reduced immune response and the difficulty in treating the infection [8]. A further unfavorable factor that proved to be of particular relevance in this pandemic is the high expression in adipose tissue of the receptor for angiotensin-converting enzyme 2 (ACE2), through which the coronavirus enters the cells [8].

During the outbreak of SARS-CoV-2, despite the following increase in the obesity rate, all non-elective surgeries were postponed, and there was the cessation of obesity management services, particularly bariatric surgery [9]. Although insufficient data are available during this pandemic, by providing substantial and sustained weight loss in most patients, bariatric surgery has been shown to reverse the negative pathological impacts of obesity. It is associated with improved survival and quality of life in patients with severe obesity [3].

For all those reasons, it is essential to implement all bariatric and metabolic surgery procedures independently from the possibility of new waves of SARS-CoV-2 [10].

At the end of the last two years of the pandemic, the aim of this umbrella review is, on the one hand, to underline the importance of seeing obesity not only as a weight problem but as an excess of fat mass and VAT, provoking a low-grade chronic inflammation, and increasing cardiovascular and severe COVID-19 risk. On the other hand, it is essential to relaunch the prioritization of bariatric surgery to prevent worsening health status in obese patients [3]. In fact, we know that COVID-19 infection will continue and will stay with us for a long.

## 2. Materials and Methods

The literature search was conducted in March 2022 via Pubmed (MEDLINE) and focused on reviews, systematic reviews, and meta-analyses. Relevant keywords to the term “bariatric surgery” OR “obesity surgery” OR “metabolic surgery” were analyzed in association with “COVID-19” OR “SARS-CoV-2” using the AND modifier. Only reviews published in peer-reviewed journals were considered to meet our inclusion criteria: articles that included specific references to bariatric and metabolic surgery and SARS-CoV-2 infection either in the title, the abstract, or the text. Original and primary studies were excluded. Three different operators independently performed the search (GLDS, RP, SG). The selection process was carried out by first analyzing the titles, then the abstracts, and finally, the full text.

## 3. Results

**Figure 1:**
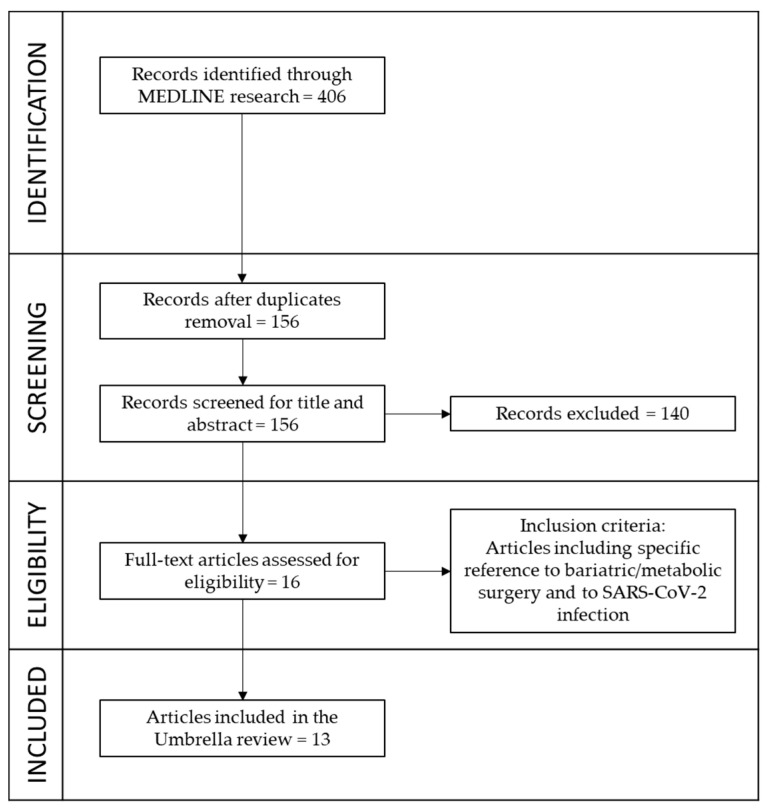
Flow diagram of study selection.

A total of 406 records resulted from the initial search; after removing duplicates, 156 articles were further assessed. Following title and abstract screening, 140 papers, and a further three after considering full texts, were excluded. Therefore, 13 studies were included in the present umbrella review. Figure 1 shows the steps of the selection process.

The characteristics of all the articles are listed in Table 1.

Of the 13 records selected:

- Five reviews are focused on strategies and management of candidates for bariatric surgery and how to restart from this pandemic;
- One review dealt with cost analysis of bariatric surgery during the pandemic;
- Three reviews highlighted the association between obesity, infectious disease, and COVID-19;
- Three reviews reported that bariatric and metabolic surgeries are protective procedures for severe clinical outcomes of SARS-CoV-2 infection; and
- One review is focused on the nutritional management of bariatric patients during the stop of bariatric surgery due to COVID-19.

## 4. Discussion

### 4.1. Obesity Pandemic

It is well established that obesity is a predisposing factor for SARS-CoV-2 infection and an adverse prognostic factor. Worldwide, individuals with a body mass index (BMI) greater than 25 kg/m^2^ (thus pre-obese) are about 39%, while those with a BMI greater than 30 kg/m^2^ (therefore obese) are 13%. The most affected countries are the United States and Europe [1].

The SARS-CoV-2 pandemic started in China in December 2019 and spread globally in early 2020. As of the paper’s writing, 464,809,377 confirmed cases since the start of the pandemic, and 6,062,536 deaths worldwide were reported. The average age of death was 75 years, with mortality rates varying from nation to nation (e.g., in Italy, 26% of patients who ended up in intensive care died; in China’s Hubei province, where the pandemic began, only 2.3%) [18].

Recent studies investigating the relationship between respiratory viruses and obesity have recognized obesity as a risk factor for a higher likelihood of hospitalization, intensive care unit admission, and mortality. Patients with severe obesity, with BMI above 40 kg/m^2^, who contracted SARS-CoV-2 infection were also found to have a higher rate of hospitalization than normal-weight subjects [4].

The pandemic of obesity has grown exponentially in recent years. In this context, obesity deserves to be considered a “social disease” and not just a disease of the individual, reflecting the profound influences of the many environmental and socioeconomic factors that condition eating habits and lifestyles [19].

During the first lockdown in Italy (9 March 2020 to 4 May 2020), changes in eating habits and lifestyles were evident. Therefore, issues related to unbalanced diets, along with the near cessation of physical activity, were exacerbated, and both of these factors worsened the obesity pandemic [14].

The procrastination of bariatric surgery over the past two years has generated a delay in obesity treatment, creating severe consequences given the high likelihood of generating new associated comorbidities or even leading to death. It is important to consider that high-stress levels, secondary to the COVID-19 pandemic, can exacerbate emotional symptoms, increasing impulsivity and triggering episodes of binge eating. Moreover, lockdown measures often led to increased food intake and decreased physical activity, increasing the risks of morbidity and mortality from pre-existing obesogenic conditions. Still, as we know, obesity itself increases the risk of many diseases, including type II diabetes, hypertension, dyslipidemia, nonalcoholic hepatic steatosis, cardiovascular and cerebrovascular diseases, cancers, osteoarthritis, and, to date, COVID-19. Patients with morbid obesity are more vulnerable to COVID-19 virus infection than the rest of the population. Patients with severe obesity have more associated comorbidities, and this group is at a much higher risk of death due to complications generated by SARS-CoV-2 infection [20]. Several studies indicate that obesity, diabetes, hypertension, and related cardiovascular conditions worsen clinical outcomes of coronavirus disease 2019 (COVID-19), including higher rates of hospitalization, need for mechanical ventilation, and mortality [15,21].

Obesity is known to negatively affect respiratory function by leading to reductions in ventilatory capacity and respiratory drive. Indeed, the accumulation of fat deposits in the chest and abdominal cavity observed in central obesity and excess visceral fat negatively affect chest wall and lung compliance. Patients with obesity are, therefore, more prone to infections and respiratory diseases, also due to their inflammatory state. Obesity is often associated with hypoventilation syndrome and obstructive sleep apnea syndrome [1]. A higher prevalence of obesity-induced hypoventilation syndrome is observed in patients with a BMI greater than 50 kg/m^2^. Furthermore, it is known that a 10% weight gain is associated with a 32% increase in the apnea–hypopnea index and a six-fold increase in the risk of moderate to severe obstructive sleep apnea [11]. It has been observed that overweight patients are associated with an 86% higher risk and obesity with a 142% higher risk of developing severe COVID-19 pneumonia than normal-weight patients [22].

Four phenotypes of obesity have been identified in the work of De Lorenzo et al. based on the combination of anthropometric, body composition, and metabolic parameters [6]: 1—normal weight obese (NWO), with a BMI < 25 kg/m^2^ but with a percentage of fat mass greater than 30% for women and 25% for men; 2—normal weight metabolically obese (MONW), with a BMI < 25 kg/m^2^, FM > 30% for women or 25% for men and with Metabolic Syndrome (MS); 3—metabolically healthy obese (MHO) (with BMI > 30 kg/m^2^, FM > 30% for women and 25% for men, without MS); 4—metabolically unhealthy obese (MUO) (with BMI > 30, FM > 30% and 25% with MS). In addition to these profiles related to the metabolic profile, it is also worth mentioning osteosarcopenic obesity, characterized by the concomitant presence of obesity, sarcopenia, and osteopenia or osteoporosis.

The condition of obesity is characterized by chronic low-grade inflammation with over-expression of inflammatory molecules and markers, such as C-reactive protein (CRP), interleukin 1 (IL-1), and interleukin-6 (IL-6). Tumor Necrosis Factor Alpha (TNF-alpha), and other adipose tissue cytokines, also named “adipokines,” can alter vascular and metabolic homeostasis, regulating target organs through the circulatory system. This mechanism underlies diseases that are often associated with obesity, such as type 2 diabetes, Alzheimer’s disease, and cardiovascular disease [23]. Now it is known that these comorbidities interfere negatively with COVID-19. Chronic low-grade inflammation, which characterizes obesity, exacerbates eventual SARS-CoV-2 infection.

It was demonstrated that IL-6 is responsible for the good maintenance of energy balance and the amount of body fat. Therefore, polymorphisms affecting this interleukin, such as −174 G/C, are capable of making changes in metabolism and energy homeostasis. This polymorphism appears to be associated with increased obesity. It is disadvantageous in terms of longevity [24]. Subjects carrying the polymorphism in the IL-6 promoter at position −174 G/C appear to have fewer results from bariatric surgery than noncarriers. They may have more damaging effects on bone [24]. The benefits of weight loss on obesity complications, insulin resistance, and systemic inflammation are well documented, especially after bariatric surgery [25].

Patients with a history of bariatric surgery also appear to be at potentially higher risk of COVID-19 vaccine breakthrough. These complications may be due in part to a predisposition to multiple comorbidities, typical of obesity, and a micronutrient deficiency due to malabsorption may play a crucial role [26]. Although it has been observed that higher BMI is associated with lower Ab titres in response to COVID-19 vaccine, the serological response is increased by an adjusted OR of 5.34 in patients with a history of bariatric surgery, compared to patients without a history of bariatric surgery [27].

In this context, it is appropriate to evaluate different strategies for treating obesity, including bariatric surgery (to be considered for patients with obesity with BMI > 35 in the presence of related comorbidities and patients with obesity with BMI > 40). Significantly reducing adipose tissue results in a marked reduction in the inflammatory state [28,29].

### 4.2. Double Pandemic: Obesity and COVID-19

There is a link between pandemics and subsequent increases in obesity rates. The culture of isolation, food-seeking behavior changes, and sedentary household activities could further worsen the obesity pandemic. The interruption of obesity management services, including bariatric surgery, during the SARS-CoV-2 pandemic further exacerbated the situation. Therefore, it is useful to know how individuals with obesity cope with this viral pandemic so we can redouble our efforts to combat obesity and its comorbidities. Obesity and age are emerging as two independent risk factors for the susceptibility and severity of COVID-19. Indeed, in older adults, immune senescence is observed, leading to greater susceptibility and more severe complications than in younger individuals. Aging leads to a deterioration in the function of the acquired and innate immune system, also linked to a decrease in the function and number of T-cells, and altered immunoglobulin levels and cytokine production [30].

Previous viral pandemics have shown that obesity, particularly severe obesity (BMI > 40 kg/m^2^), and being elderly are associated with an increased risk of hospitalization, critical care hospitalization, and death [31]. Patients ≥ 65 years, as well as the ones with severe obesity, in fact, represent one of the most important groups with a higher risk of serious diseases, such as COVID-19. Furthermore, it is observed that in patients in the COVID-19 intensive care unit, those aged ≥ 75 years and those with a body mass index (BMI) > 40 kg/m^2^ partly represent the highest risks [30]. It is also known that all age groups can be infected with SARS-CoV-2. The median age of hospitalized cohorts ranged from 47 to 63 years, and the median age of death from the disease was 75 years. Age and obesity are, therefore, negative prognostic factors in COVID-19. It is also observed that the prevalence of obesity in men and women increases with age. However, although in men a correlation between worse COVID-19 outcomes and reduced testosterone due to age and obesity has been hypothesized, no direct correlations between age, obesity, and COVID-19 seem to have been investigated [1].

In many countries, during the ongoing COVID-19 pandemic, bariatric surgery has been evaluated only as an elective intervention that can be postponed with minimal adverse consequences. This approach, however, neglects severe obesity as a dangerous and life-limiting disease and fails to recognize the intertwined double pandemic of COVID-19 and obesity [4].

Manifestations of COVID-19 range from asymptomaticity to death, across a broad clinical spectrum, from fever, cough, myalgia, fatigue, dyspnea, progressive respiratory failure, bowel symptoms, pneumonia, acute respiratory distress syndrome (ARDS), myocarditis, and organ failure, to death. Obesity affects respiratory function through various mechanisms, such as lung restriction, ventilation–perfusion mismatch, and respiratory muscle fatigue. In addition, increased abdominal pressure and limited chest expansion reduce ventilatory capacity. These complications increase the risk of obesity hypoventilation syndrome, particularly in those with severe obesity [32]. Many patients with obesity and associated comorbidities can worsen the clinical outcomes of SARS-CoV-2 infection. Then, the mechanical effects of excess fat on the function of the chest wall, diaphragm, and lung may contribute to the severity of the clinical status of patients with obesity after the development of COVID-19 pneumonia [22,33].

The presence of comorbidities aggravates the course of COVID-19. Obesity is a pro-coagulant and pro-inflammatory state that increases the risk of thrombosis, cytokine level, and oxidative stress response. It can impair the innate and adaptive immune response to infection [3]. The proinflammatory alteration associated with obesity is an important risk factor for acute lung injury. In addition, low-grade inflammation increases leptin (proinflammatory adipokine) and decreases adiponectin levels (anti-inflammatory adipokine) [8]. Adiponectin deficiency has also increased lung inflammation and reduced apoptotic cell clearance. Interestingly, adiponectin levels in MHO individuals are higher than in those with impaired metabolic health (MUO). The latter group is predisposed to an increased risk of pneumonia and a worse outcome of COVID-19 [1].

Adipose tissue expresses various receptors and enzymes necessary for SARS-CoV-2 to be able to infect. ACE2, the functional receptor for SARS-CoV and SARS-CoV-2, is highly expressed in adipose tissue, particularly in visceral adipose tissue (VAT), compared with subcutaneous adipose tissue (SAT). Importantly, its expression is increased in adipocytes of patients with obesity and diabetes. Therefore, it has been hypothesized that the adipose tissue of obese patients may serve as a target organ of SARS-CoV-2 and as its viral reservoir. This reservoir could act as an accelerator that results in high inflammation, fueling an excessive and counterproductive immune response, the so-called cytokine storm, damaging tissues and causing multi-organ failure, which is the most serious complication of COVID-19 [34]. Among cytokines, it is important to mention TNF-α, a pyrogenic cytokine released in the acute phase of inflammation by macrophages and immune cells. It is known that TNF-α expression in lung epithelial cells is higher during influenza and viral infections. In patients with COVID-19 and obesity, high serum levels of IL-6 and TNF-α are negatively associated with T cells. In contrast, T-cell levels are restored by reducing concentrations of both IL-6 and TNF-α. These results suggested that these cytokines could be important targets of anti-COVID-19 therapies [4].

IL-6 is also a pro-inflammatory cytokine produced by adipose tissue. Therefore, this endocrine cytokine might be important in regulating the host response during acute infection. Several articles have described the essential role of IL-6 in generating an adequate immune response during different types of viral infection in the lung tract. Others link this cytokine to exacerbation of the viral disease, supporting the hypothesis that up-regulation of IL-6 during viral infections may promote virus survival and clinical disease exacerbation [1]. IL-6 has a pleiotropic function and is produced in response to tissue damage and infection. In particular, at the lung level, the proliferation of innate and adaptive immune cells is strongly influenced by this cytokine [23].

Another aspect that should not be underestimated is the hypothesis that there may be a positive correlation between infectivity and weight gain. There is a paucity of evidence on the infectivity of individuals with obesity during the COVID-19 pandemic. The virus is spread by human-to-human transmission through droplets, aerosolized particles, and direct contact with an incubation time ranging from 2 to 14 days [35]. Some studies would seem to show that obese patients are potentially more contagious than lean individuals in case of viral infections. People with obesity who contract the influenza virus remain contagious 42% longer than people without obesity. Indeed, it was observed that obesity increases the duration of viral shedding and delays the ability to produce interferons, allowing further viral RNA replication and increasing the chances of new, more virulent viral strains emerging [22].

Rapid weight loss after bariatric surgery ameliorates the multiple co-morbidities of obesity. Consequently, it is plausible to argue that early intervention in obesity can help minimize the effects of the disease in the event of a new SARS-CoV-2 outbreak. Furthermore, solid evidence in the literature indicates that a larger obese population increases the likelihood of a more virulent viral strain, prolongs the spread of the virus throughout the community, and ultimately may increase overall pandemic mortality rates [36]. In the meantime, patients should be optimized for surgery by ensuring that their weight and metabolism are controlled through diet, lifestyle, and pharmacological measures. Surgery should be accelerated for patients with morbid obesity who do not respond to these conservative measures [14]. However, even small amounts of short-term weight loss can show substantial metabolic benefits. Some of these positive events happen even few days after surgery, i.e., reduction of hyperglycemia. This is especially important during the COVID-19 pandemic; weight loss must be encouraged as a public health intervention. Healthcare providers should discuss weight loss goals and methods with the patients. Obesity management and bariatric surgery teams must be advocates for their patients during these difficult times. The risk will be that patients’ needs will be further ignored because of the public perception that obesity is still a choice and not a disease [37].

### 4.3. Benefits of Bariatric Surgery in General and Respect to SARS-CoV-2 Infection

The SARS-CoV-2 pandemic has had devastating effects on obesity, first because it has created an environment conducive to the development of obesity and second because it has reduced access to treatment pathways for obesity and related conditions. In particular, the pandemic has reduced access to bariatric surgery entry and follow-up pathways [4].

The first case of human-to-human transmission in Italy was on 21 February 2020. After that, cases increased rapidly, and several restraining measures and hospital bed reallocations were necessary. By the end of February 2020, almost all non-oncologic elective surgeries had been cancelled to make way for the flow of patients with COVID-19. Bariatric surgery, considered elective surgery, was suspended and gradually, but still partially, restarted from 4 May 2020, when epidemiological data showed the effectiveness of the restraining measures undertaken [2]. Following waves brought to a new reduction in bariatric surgery, with negative consequences for the patients on the waiting list [17].

Bariatric and metabolic surgery results in massive weight loss in most patients and reduces the negative effects of obesity on body health and respiratory mechanics. In patients with severe obesity, body weight reduction following bariatric surgery results in improved survival and quality of life. In addition, some studies show that weight loss from bariatric surgery is protective against severe SARS-CoV-2 infection [3]. Delaying bariatric surgery in patients with morbid obesity may cause the worsening of obesity-associated diseases.

A systematic review on obesity and COVID-19 showed a significant association between obesity and higher SARS-CoV-2 infectious risk (odds ratio [OR] 1.5, *p* < 0.001), higher risk of severe SARS-CoV-2 infection (OR 3.13, 95% *p* = 0.005) and higher mortality (OR 1.36, 95% *p* = 0.006) [38].

A French study of 124 ICU patients reports that obesity and morbid obesity affected 47.6% and 28.2% of patients who became ill with COVID-19. Supporting this evidence are data from previous outbreaks attributable to different viral agents that also show a correlation between obesity and the severity of clinical manifestation of viral disease [39]. The largest prospective observational cohort study conducted in Europe with near real-time data collection and analysis, using a pre-approved questionnaire adopted by the World Health Organization, conducted in 166 hospitals in the United Kingdom between 6 February and 18 April 2020, and involved 16,749 people with COVID-19, reported that obesity was associated with higher mortality. In addition, in a cohort study of critically ill patients with confirmed SARS-CoV-2 infection admitted to the intensive care unit in Brescia (Lombardy, Italy) between 2 March 2020 and 13 March 2020, 31% of patients were obese, and an additional 58% were overweight [2].

In this context, bariatric surgery is essential in reducing obesity-related complications of SARS-CoV-2 infection. Specifically, a multicenter study showed that patients who had undergone bariatric surgery at least 12 months earlier developed COVID-19 in less severe forms than obese patients not treated with bariatric surgery [16].

A meta-analysis of observational studies published in December 2020 showed that mortality and hospitalization rates in patients with SARS-CoV-2 infection were lower in obese patients who had undergone bariatric surgery than in those who had not received such treatment. In this meta-analysis, in obese patients with SARS-CoV-2 infection without a history of bariatric surgery, the risk of mortality was 133 per 1000, while in patients with a history of bariatric surgery, it was 33 per 1000 (OR 0.22, 95% CI 0.19–0.26). In obese patients with SARS-CoV-2 infection without a history of bariatric surgery, the hospitalization rate was 412 per 1000. In patients with a history of bariatric surgery, it was 164 per 1000 patients (OR 0.28, 95% CI 0.12–0.65). Furthermore, bariatric surgery shows benefits by counteracting the negative effects of age on COVID-19 patients [3].

In a self-controlled case series study on patients undergoing bariatric surgery, the risk of contracting respiratory infections was significantly reduced during the first 12 months after bariatric surgery. Therefore, bariatric surgery may help minimize the effects of SARS-CoV-2 infection in obese individuals even shortly after surgery [2].

Few studies have investigated the effects of bariatric surgery in patients with morbid obesity who contracted COVID after surgery. An Italian multicenter retrospective study investigated the impact of bariatric surgery in patients between January and February 2020, when there were no SARS-CoV-2-containing measures. During this period, 840 bariatric surgeries were performed in the eight Italian centers surveyed. In May 2020, patients were contacted by telephone; only five of the 840 had contracted SARS-CoV-2. In particular, two had mild symptoms, three were hospitalized, and none died. The rate of postoperative complications was similar to that of 836 patients who underwent bariatric surgery the previous year during the same period [2].

Afterward, restraining measures were taken, and elective surgeries, including bariatric surgery, were postponed. During the epidemic peak, patients were operated on for cancer surgery or underwent emergency surgery between March and April 2020 in northern Italy. Still, when differentiated pathways between COVID and non-COVID patients had already been created, they did not have a higher morbidity and mortality rate compared with the same months in the previous year. This would prove that the separation of COVID/non-COVID pathways is safe.

### 4.4. Economic Aspects

In addition to the purely medical aspect, in a world where resources are starting to become scarce, an analysis of the costs of obesity and COVID-19 deserves attention.

Obesity determines an economic burden for the individual and health care systems. The costs of obesity can be divided into direct and indirect costs. Direct costs are those associated with the treatment of obesity and its complications, while indirect costs are those associated with lost productivity and lost workdays due to disability and obesity-related physical and psychological problems [40].

In 2014, a report by the McKinsey Global Institute estimated that the economic impact on the world economy due to obesity amounts to US$2 trillion, equivalent to 2.8% of global gross domestic product (GDP) [16]. In this era, when it is becoming increasingly important to pay attention to the distribution of health care investments and beyond, it seems clear that proper care of patients with obesity is necessary to derive an economic benefit as well. At this moment in history, this issue deserves increasing attention because obesity and its associated costs are bound to increase unless appropriate measures are taken to combat it.

COVID-19, in addition to having a huge impact on health, has resulted in huge losses in economic terms. Again, there were direct costs related to medical expenses and indirect costs related to lost productivity (lost workdays due to illness and isolation and lost productivity related to long-term complications of the infection). The lockdown has resulted in an economic contraction and the closure of many manufacturing activities. According to the International Monetary Fund (IMF), there has been a 3.3% contraction in global GDP in 2020 [16].

In this context, a strategy must be found that combines the possibility of offering the best care to the patient with obesity with the option of economic savings. The implementation of bariatric surgery is one of the possible strategies to achieve this goal.

Bariatric surgery remains the gold standard treatment of morbid obesity and has been widely shown to improve associated comorbidities. Although bariatric surgery has high costs, a 2019 meta-analysis, which only considered the direct costs associated with obesity, demonstrated a lifetime cost advantage over conventional weight loss methods [41].

A 2018 systematic review also supports the evidence that bariatric surgery is cost-effective [42]. This benefit is related to improving or resolving the obesity and related conditions. Specifically, weight loss resulting from bariatric surgery reduces addiction to drug therapy. Also, it is understood how reducing the need for pharmacological therapy for diabetes, hypertension, dyslipidemia, and other chronic conditions associated with obesity decreases the economic burden on the healthcare system [43]. A cost-utility analysis conducted in the United Kingdom supports that bariatric surgery determines an annual saving of US$2689 per patient and an increase of 0.8 life-years and 4.0 quality-adjusted life-years [16].

### 4.5. How to Restart

The SARS-CoV-2 pandemic since the first half of 2020 has had a significant impact worldwide. A large number of patients with SARS-CoV-2 infection poured into emergency departments since the beginning of March 2020, and the emerging need for intensive care beds has strained the Health Care System around the world, literally paralyzing operating rooms [13].

During the outbreak of COVID-19, announced by the World Health Organization as a pandemic in March 2020, most non-emergency surgeries were postponed worldwide due to intraoperative risks of viral contagion among patients and healthcare workers [12].

As well as for the non-emergency surgical treatment of many other conditions, the surgical treatment of obesity and its complications was postponed throughout the state of emergency because they were considered “elective” procedures, where elective is defined as “relating to, being or involving a nonemergency medical procedure and particularly a surgical procedure planned in advance and not essential to the survival of the patient”. As electives, they were also considered the last to be restarted. The American Society for Metabolic and Bariatric Surgery (ASMBS) has defined metabolic and bariatric surgery as NOT elective and the best treatment for people with severe obesity, a life-threatening and life-limiting condition [10].

Several studies show that bariatric surgery improves both survival and obesity-related comorbidities. It can also make long-term changes in metabolism, improving long-term health and quality of life [44].

Therefore, it should be placed on waiting lists to ensure a maximum delay of 6 months, as it is for other benign surgical interventions. Delay in bariatric surgery can have fatal consequences; in fact, up to 50% of patients develop new comorbidity while on the waiting list, and 1.5% of patients die while waiting for surgery. This underscores the need for the resumption of bariatric surgery contextually with the significant current decrease in the number of hospitalizations of COVID-19 patients and the newfound availability of sufficient resources and safety to restart surgeries in patients with the benign disease [11].

Despite the protection provided by vaccines, it remains essential to follow precautions to limit the number of infections. It is certain, however, that from a patient-centered and public health perspective, it is critical to resume metabolic and bariatric surgery. The resumption of elective surgery requires careful planning and the introduction of new preventive measures to protect both patients and healthcare workers [45]. It will be necessary to maintain ‘COVID-19-free’ circuits within the hospital to ensure maximum safety, conduct periodic checks of healthcare workers, identify possible cases early, and avoid performing elective surgery in infected patients [46].

To safely perform bariatric surgery, it is necessary to ensure a clean ‘COVID-free’ circuit in the different places of the obesity patient pathway: outpatient clinics, wards, operating rooms, and recovery rooms. Therefore, it is essential to conduct daily symptom screening in patients to detect possible SARS-CoV-2 positive patients at an early stage. Instead, the post-bariatric surgery follow-up protocol may be similar to the pre-pandemic protocol. Still, it will need to be implemented with the possibility of remote care supported by telemedicine [47].

Due to resource and space constraints, the return to bariatric and metabolic surgery has been gradual, and not yet completely accomplished. Therefore, priority criteria need to be established to give order to interventions. In obese patients, the risk of developing further complications if surgery is postponed varies among individuals depending on the type of obesity, severity, and associated comorbidities. Therefore, there is a need to develop strategies to limit and mitigate the harm suffered by patients [11].

It must be emphasized that new drugs and the ones that are going to come in the market can positively treat morbid obese persons in preparation for bariatric surgery.

These strategies should include nonsurgical interventions to optimize metabolism and weight control in patients awaiting surgery, telemedicine protocols for postoperative surveillance, and using appropriate criteria for triaging surgical candidates during a period of still reduced capacity for elective surgery.

Considering the factors contributing to morbidity and mortality in obesity and type 2 diabetes, surgical prioritization should be based on disease-specific considerations. For example, for patients with type 2 diabetes, priority could be given to patients at increased risk of morbidity and mortality indicated by poor glycemic control, insulin use, cardiovascular disease, albuminuria, chronic kidney disease, nonalcoholic steatohepatitis, or multiple cardiometabolic comorbidities. In general, priority could be given to surgery in patients with more than five years of diabetes to limit the risk of reduced treatment efficacy [48], and to those with major eating behavior disorders.

As reported by the experience of other countries, even in Italy, the resumption of elective bariatric surgery aims at minimizing the risks to patients and healthcare personnel. The patient should arrive at the hospital 24–48 h before admission. Outside the hospital, healthcare personnel should swab the patient and, in case of positivity, should cancel the surgery [49]. These procedures follow international and national Ministry of Health guidelines for pre-hospitalization screening of patients undergoing elective surgery, so bariatric surgery centers must follow these guidelines [50].

## 5. Conclusions

Bariatric surgery has been shown to treat obesity and its comorbidities, improving health, quality of life, and long-term survival. It also results in economic benefits for the individual and the overall health and financial system.

Some authors suggest that COVID-19 should now be added to the long list of obesity comorbidities that can be mitigated by bariatric surgery [33].

All these diseases reduce the quality of life and increase psychosocial dysfunction, morbidity, and mortality. Even without taking COVID-19 into account, obesity and related comorbidities reduce life expectancy by about 5–20 years [42]. As the COVID-19 pandemic continues, health policies and interventions must recognize obesity as a modifiable disease as well as a predisposing factor for SARS-CoV-2 infection.

Therefore, care pathways for patients with obesity in the COVID/post-COVID era should be revitalized. The need to fully restart bariatric surgery and its related treatment finds a place in this context. It seems clear that the concept of elective surgery, which is attributed to bariatric surgery, needs to be reformulated.

## Figures and Tables

**Table 1:** Summary of characteristics of included studies in most recent reviews, systematic reviews, and meta-analyses.

Author	Title	Type of Paper	Date	Finding
Rubino, F.; et al. [11]	Bariatric and metabolic surgery during and after the COVID-19 pandemic: DSS recommendations for management of surgical candidates and postoperative patients and prioritisation of access to surgery	Review	7 May 2020	Review by experts of the “Diabetes Surgery Summit consensus conference” providing guidance for the management of patients while surgery is delayed for COVID-19 and for postoperative surveillance. In particular, strategies to prioritise bariatric and metabolic surgery candidates on the basis of the diseases that are most likely to be ameliorated postoperatively.
Erol, V.; et al. [12]	Recommendations for bariatric and metabolic surgical operations during the COVID-19 pandemic in Turkey.	Review	20 May 2020	The aim of this review was to determine the pre-peri and post-operative periods of bariatric surgical requirements during the COVID-19 pandemic according by the Turkish Society for Metabolic and Bariatric Surgery.
Executive Council of American Society for Metabolic and Bariatric Surgery, [10]	Safer through surgery: American Society for Metabolic and Bariatric Surgery statement regarding metabolic and bariatric surgery during the COVID-19 pandemic	Review	6 June 2020	Metabolic and bariatric surgery is life-saving and life-changing surgery, and especially during this pandemic COVID-19 emerged as the most recent of many diseases in which underlying obesity worsens the prognosis.
Marinari, G.M.; et al. [2]	Bariatric and metabolic surgery during COVID-19 outbreak phase 2 in Italy: why, when and how to restart	Review	9 June 2020	Review on the Italian experience for a safe restart of elective laparoscopic bariatric surgery thanks to the deliverance of transparent information to the patients and the introduction of the COVID-19 protocol concerning patients and health-professionals protection.
Sanchez Santos, R.; et al. [13]	Obesity and SARS-CoV-2: considerations on bariatric surgery and recommendations for the start of surgical activity.	Review	18 June 2020	This document contains the main recommendations for bariatric surgery programs in Spain from the point of view of safety, preparation of the bariatric patient and follow up during the SARS-CoV- pandemic.
Kwok, S.; et al. [1]	Obesity: A critical risk factor in the COVID-19 pandemic	Review	7 August 2020	Review highlighting potential mechanisms by which obesity may influence adverse outcomes from COVID-19, including chronic inflammation, impairment of respiratory function and pulmonary perfusion, immune dysregulation, metabolic and vascular complications; people with severe obesity must be considered a vulnerable group for COVID-19.
Aminian, A.; et al. [3]	Association of Bariatric Surgery with Clinical Outcomes of SARS-CoV-2 Infection: a Systematic Review and Meta-analysis in the Initial Phase of COVID-19 Pandemic.	Systematic Review and Meta-analysis	30 December 2020	Prior bariatric surgery is associated with a lower rate of mortality and hospital admission in patients with obesity and infected with SARS-CoV-2.
Zakka, K.; et al. [4]	SARS-CoV-2 and Obesity: “CoVesity”—a Pandemic Within a Pandemic	Systematic Review	22 January 2021	Overview on the link between obesity, a pandemic in itself, as an independent factor for having a worse outcome among COVID-19 patients; the dual pandemic—CoVesity—will have a detrimental outcome in the short, medium, and long term.
De Amicis, R.; et al. [14]	Patients with Severe Obesity during the COVID-19 Pandemic: How to Maintain an Adequate Multidisciplinary Nutritional Rehabilitation Program?	Review	19 March 2021	The adoption of new strategies, as telemedicine, is necessary to guarantee a continuous multidisciplinary nutritional program to control the severity of SARS-CoV-2 infection in obese patients.
Gupta, R.; et al. [15]	Impact of COVID-19 on the outcomes of gastrointestinal surgery	Review	29 April 2021	Review of recent studies reporting the outcomes of various gastrointestinal surgeries in the COVID-19 pandemic era including bariatric surgery; bariatric surgery can be offered to the obese patients if the healthcare resources are present and appropriate measures are taken to prevent perioperative transmission of SARS-CoV-2 infection.
Liu, D.; et al. [16]	Role of bariatric surgery in a COVID-19 era: a review of econimic costs	Review	17 July 2021	Review on the need to the resumption of bariatric services as quickly as possible because of the significant additional medical and economic benefits that it provides.
Dickey, J.; et al. [17]	Homeostasis Disrupted and Restored—A Fresh Look at the Mechanism and Treatment of Obesity During COVID-19	Review	27 August 2021	Weight loss during COVID-19 era decrease morbidity and mortality in obese patients, and bariatric surgery should not be postponed.
Pugliese, G.; et al. [8]	Obesity and infectious diseases: pathophysiology andepidemiology of a double pandemic condition	Review	21 January 2022	Obesity is related directly to a higher risk of contracting different infectious diseases, as well as experiencinga more severe course of COVID-19 with increased mortality rates.

## Data Availability

Not applicable.
